# *Ulva prolifera* green-tide outbreaks and their environmental impact in the Yellow Sea, China

**DOI:** 10.1093/nsr/nwz026

**Published:** 2019-03-19

**Authors:** Yongyu Zhang, Peimin He, Hongmei Li, Gang Li, Jihua Liu, Fanglue Jiao, Jianheng Zhang, Yuanzi Huo, Xiaoyong Shi, Rongguo Su, Naihao Ye, Dongyan Liu, Rencheng Yu, Zongling Wang, Mingjiang Zhou, Nianzhi Jiao

**Affiliations:** 1 Key Laboratory of Biofuels, Shandong Provincial Key Laboratory of Energy Genetics, Qingdao Institute of Bioenergy and Bioprocess Technology, Chinese Academy of Sciences, Qingdao 266101, China; 2 Institute of Marine Microbes and Ecospheres, State Key Laboratory of Marine Environmental Science, Xiamen University, Xiamen 361101, China; 3 College of Marine Ecology and Environment, Shanghai Ocean University, Shanghai 201306, China; 4 Institute of Marine Science and Technology, Shandong University, Qingdao 266237, China; 5 Department of Oceanography, Dalhousie University, Halifax, B3H 4R2, Canada; 6 National Marine Hazard Mitigation Service, State Oceanic Administration, Beijing 100194, China; 7 College of Chemistry and Chemical Engineering, Ocean University of China, Qingdao 266100, China; 8 Yellow Sea Fisheries Research Institute, Chinese Academy of Fishery Sciences, Qingdao 266071, China; 9 State Key Laboratory of Estuarine and Coastal Research, East China Normal University, Shanghai 200062, China; 10 Key Laboratory of Marine Ecology and Environmental Sciences, Institute of Oceanology, Chinese Academy of Sciences, Qingdao 266071, China; 11 Key Laboratory of Science and Engineering for Marine Ecology and Environment, the First Institute of Oceanography, State Oceanic Administration, Qingdao 266061, China

**Keywords:** green tides, *Ulva prolifera*, outbreak mechanisms, eco-environmental effects, Yellow Sea

## Abstract

The *Ulva prolifera* green tides in the Yellow Sea, China, which have been occurring since 2007, are a serious environmental problem attracting worldwide attention. Despite extensive research, the outbreak mechanisms have not been fully understood. Comprehensive analysis of anthropogenic and natural biotic and abiotic factors reveals that human activities, regional physicochemical conditions and algal physiological characteristics as well as ocean warming and biological interactions (with microorganism or other macroalgae) are closely related to the occurrence of green tides. Dynamics of these factors and their interactions could explain why green tides suddenly occurred in 2007 and decreased abruptly in 2017. Moreover, the consequence of green tides is serious. The decay of macroalgal biomass could result in hypoxia and acidification, possibly induce red tide and even have a long-lasting impact on coastal carbon cycles and the ecosystem. Accordingly, corresponding countermeasures have been proposed in our study for future reference in ecosystem management strategies and sustainable development policy.

## INTRODUCTION

**Figure 1.**
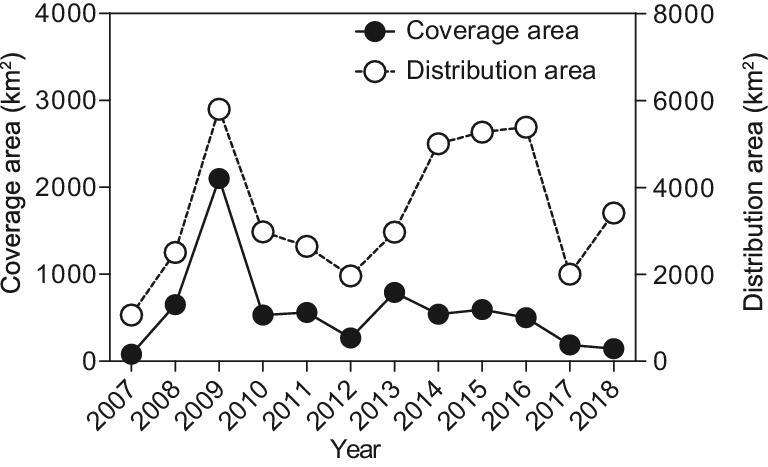
Variation of the distribution and coverage areas of the *U. prolifera* green tides from 2007 to 2018 (http://www.soa.gov.cn/zwgk/hygb/zghyzhgb/).

Massive green tides, which are mainly caused by *Ulva prolifera* (*U. Prolifera*) have successively occurred for 12 years (2007–18) in the Yellow Sea off the coasts of Jiangsu and Shandong Province, China [1,2]. In particular, during 2008, massive *U. prolifera* fronds floated into the coasts of Qingdao, posing a great challenge to the local government owing to the upcoming Olympic Sailing Regatta to be held in Qingdao. The direct economic losses caused by that macroalgal bloom were as high as 1.3 billion RMB [3]. Every spring (from mid-April to early May) since 2007, macroalgal blooms have initially occurred along the Jiangsu coast with small-scale floating algae, then migrated northward along the coast of the southern Yellow Sea driven by monsoons and ocean currents, accumulating in the near-shore waters of the Shandong Peninsula in June and July, and then declined gradually [4]. With the annual coverage area of over 20 000 km^2^, *U. prolifera* bloom in the Yellow Sea is the largest green-tide event in the world so far (Fig. 1) [5].

Research and monitoring over 10 years have shown that the outbreak mechanisms of *U. prolifera* green tides can be attributed to multiple factors. Remote sensing data have demonstrated that *U. prolifera* green tides originally occurred along the Jiangsu coast [6–13] and gene sequencing data have confirmed that the *U. prolifera* growing in *Porphyra* mariculture areas along the Jiangsu coast is the same species as that found in the Yellow Sea [14]. It also reveals that the unique mud flats for cultivation of *Porphyra* are favourable habitats for *U. prolifera* and that more than 20 000 ha of mariculture rafts are good substrata for the propagation of *U. prolifera* [2,5,15]. Moreover, the *Porphyra* mariculture area is characterized by high nutrient levels (i.e. NO_3_–N >10 μM, PO_4_–P > 0.3 μM and SiO_3_–Si >15 μM), which could almost meet the nutrient demands of *U. prolifera* for its rapid growth [16]. In addition, a large number of micropropagules of *U. prolifera* that germinate on the mariculture rafts in seawater or sediments after the *Porphyra* harvest grow into new fronds as the temperature increases, thus serving as the original species of green tides occurring in the following spring [17,18].

As indicated by morphology and genomics, *U. prolifera* is considered to be the dominant green-tide-forming seaweed in the Yellow Sea [19–21]. Other species of the *Ulva* genus, such as *U. intestinalis, U. linza, U. compressa* and *U. clathrate*, have also been observed in *Porphyra* mariculture areas along the Jiangsu coast [22]. However, *U. prolifera* can outcompete others owing to its higher nutrient-uptake rate, faster growth and stronger reproduction capacity [23]. The proportion of *U. prolifera* among the raft-attached macroalgae usually increases with *Porphyra* growth. When the attached macroalgae are discarded into seawater after the *Porphyra* harvest, the proportion of *U. prolifera* in floating algae can increase from 63 to 91% within 24 hours under optimal environmental conditions [2,15]. Moreover, besides its own physiological advantages of *U. prolifera*, appropriate physicochemical conditions (e.g. favourable temperature, salinity and light intensity), southeastward monsoons, etc., can all contribute greatly to the occurrence of green tides [5].

As a matter of fact, the outbreak mechanisms of green tides in the Yellow Sea are far more complicated than had been thought before. In the past decade, various attempts have been made to prevent the annual occurrence of large-scale green tides but were unsuccessful. However, in 2017, *U. prolifera* green tides decreased abruptly in the Yellow Sea, which aroused further curiosity and attention. Why did *U. prolifera* green tides first occur in 2007 and decrease remarkably in 2017? What have not been identified so far that are closely related to the green-tide outbreak? We are gradually unravelling the reasons behind this phenomenon.

**Figure 2.**
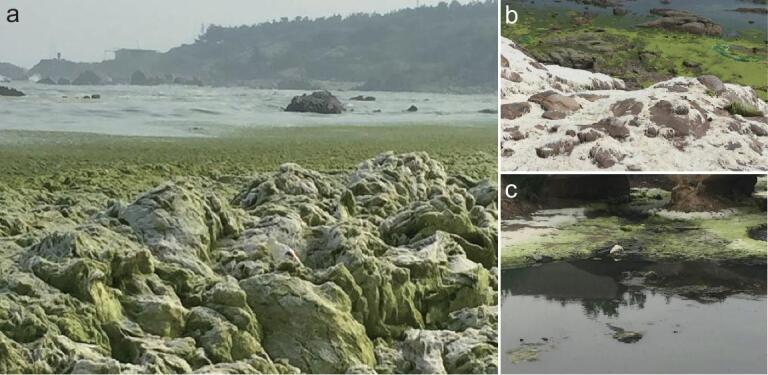
Environmental impacts of the *U. prolifera* green tides as seen from the situation in the Qingdao coasts in July, 2016. (a) Coastlines covered with *U. prolifera* fronds accumulated by natural tidal movements; (b) *U. prolifera* fronds bleached under sunlight; (c) decay of *U. prolifera* fronds.

Massive macroalgal blooms can have a negative impact on the coastal environment; for example, they can seriously upset the balance of coastal ecosystems, affect air–sea exchanges and even cause the death of cultured organisms, such as sea cucumber and shellfish, in the Yellow Sea [24]. During the late development stage of green tides, the decomposition of massively accumulated fronds can degrade seawater quality and cause foul odours (Fig. 2), both of which can seriously interfere with local tourism and coastal mariculture in the Shandong Peninsula. In addition, the massive macroalgal bloom can have a long-term impact on marine biogeochemical cycles.

This review is aimed at a systematic analysis of multiple factors in facilitating the outbreaks of *U. prolifera* green tides as well as the impact on the environment of the Yellow Sea. Based on the recognition of the outbreak mechanisms of green tides and their following consequences, we proposed corresponding countermeasures to prevent the frequent occurrence of green tides in the future.

## THE BLOOMING PROCESS OF *ULVA PROLIFERA* GREEN TIDES IN THE YELLOW SEA

The *U. prolifera* green tide is a trans-regional disaster in the Yellow Sea, China. These macroalgal blooms usually originate in the coastal areas of the southern Yellow Sea during mid-April to early May, and start as small patches of free-floating green algae. Subsequently, the free-floating algal patches move northward and northeastward, driven by seasonal monsoons and ocean currents. During the migration period, *U. prolifera* grows fast and accumulates gradually. In late May, large floating *U. prolifera* aggregates into long large floating mats. Nearly all the floating macroalgae are observed in linear bands ranging from hundreds of metres to tens of kilometres in the open sea area. In mid-June, floating *U. prolifera* accumulates with a huge biomass (∼1.5 × 10^6^ tons) and covers a large area in the coastal sea of the Shandong Peninsula. Thereafter, great social and economic losses emerge due to the large algal biomass piling up on beaches and coastal waters. At the end of June, *U. prolifera* continues to grow, causing the biomass to reach as high as ∼3.5 × 10^6^ tons. In mid-July, *U. prolifera* reaches its maximum biomass of ∼1.2 × 10^7^ tons. After that, *U. prolifera* green tides begin to decline. In early August, the floating *U. prolifera* can only be sporadically observed in the coastal areas [15,25].

## REGIONAL FACTORS FOR MACROALGAL BLOOMS

Every spring from 2007, *U. prolifera* green tides have developed from small-scale floating patches into massive macroalgal blooms, which is closely related to various factors including seawater temperature, salinity, light intensity, wind direction and ocean currents in the Yellow Sea.

### Favourable temperature, salinity and light intensity

Seawater temperature has been identified as a key factor in influencing the growth of *U. prolifera* [2,15]. *Ulva prolifera* generally grows well when temperature ranges between 10 and 20°C; its biomass can increase by as much as 23% per day when the temperature exceeds 15°C, while it cannot grow when the temperature is below 10°C [26,27]. The daily average temperature ranges from 6 to 21°C along the Jiangsu coast during spring (from March to May), which is ideal for the growth of *U. prolifera* [15]. Vegetative propagation is one of the main reproduction modes for *U. prolifera*, which may contribute to the rapid growth of the fronds. The algal fragment of *U. prolifera* grows by 28% per day at 20°C and only by 3% per day at 10°C in laboratory incubations [28]. Moreover, the suitable temperature range for the release of spores and gametes of *U. prolifera* is 20–30°C [29] and the temperature for the germination of these micropropagules is 15–25°C [18,22,30]. This, to some extent, also accounts for the occurrence of annual large-scale macroalgal blooms in late April or early May. Additionally, O_2_ and CO_2_ are produced separately in photosynthesis and respiration and fill the fronds of *U. prolifera*, causing them to float in the surface water [5]. *Ulva prolifera* can have normal photosynthesis only when the temperature exceeds 10°C; the water temperature during May to June in the Yellow Sea ranges from 15 to 25°C, which is quite suitable for the floating and accumulation of *U. prolifera*. As mentioned in the introduction, *Porphyra* mariculture rafts are good substrata for the early propagation of *U. prolifera*. Considering the obvious impact of temperature on the growth of *U. prolifera*, earlier harvest of *Porphyra* in the southern Jiangsu Province, especially before the temperature reaches over 10°C in April, may reduce the biomass of *U. prolifera* fall off the rafts and thus lower the outbreak scale of green tides [31].

The impact of salinity on *U. prolifera* cannot be ignored either. Suitable salinity for the growth of *U. prolifera* has been reported to range between 8 and 32 [32]. The photosynthetic responses of *U. prolifera* to short-term salinity fluctuations indicate that *U. prolifera* is quite sensitive to salinity changes. The photosynthetic rate is significantly higher with high salinity (26–32), optimum at 32 and low salinity (<8) has adverse effects on the photosynthesis. In coastal waters of the Yellow Sea, the salinity usually varies between 28 and 31 during the spring and summer periods (from April to June), which aligns within the suitable salinity ranges for the growth of *U. prolifera* [22]. Besides its effects on growth, salinity also influences the reproduction of *U. prolifera*; for example, lower salinity (i.e. 20) promotes its sporangium formation and increases spore germination as well [33]. Furthermore, the growth of *U. prolifera* was compared under different temperatures (i.e. 15 or 22°C) and salinities (i.e. 10 or 25), the results of which indicate that, with low salinity (i.e. 10), the growth of *U. prolifera* decreases compared with high salinity (i.e. 25) [34]. All of the above results indicate that *U. prolifera* can endure drastic variability in salinity and modest salinity variations, to some extent, can benefit its growth and blooming.

Apart from temperature and salinity, light intensity also affects the physiology of *U. prolifera*, including the growth, photosynthesis, reproduction, nutrient uptake, utilization, etc. [4,35]. Light promotes algae growth, but excessive light inversely inhibits their growth. However, microalgae often have a high tolerance to light conditions [26], owing to their various photosynthetic adaptation strategies, like changing cellular pigment components or dissipating excess energy via photosynthetic quantum control (energy quenching and energy redistribution between PSII/PSI) [21]. For the green-tide-forming species *U. prolifera*, suitable light intensity to drive its growth has been reported to vary from 18 to 175 μM photons m^−2^ s^−1^ [26]. Of course, such light intensity for growth also varies with changes in other environmental factors like nutrients, salinity and temperature [21,35]. Under ammonium-enrichment conditions, its maximum photosynthetic rate can reach 370 μM O_2_ g FW^−1^ h^−1^ at the optimal light level of 120 μM photons m^−2^ s^−1^ [35]. The light intensity also affects the reproduction of *U. prolifera* by regulating the release or germination of spores and gametes. The optimal light level for the release of spores and gametes has been reported to be 240 μM photons m^−2^ s^−1^ while, for their germination, it is 120 μM photons m^−2^ s^−1^ [36]. For asexual reproduction, however, the highest reproduction rate has been found at a light intensity of 100 μM photons m^−2^ s^−1^ [28]. Furthermore, light provides energy to drive nutrient absorption by algae. *Ulva prolifera* was observed to have optimal ammonium absorption at a light level of 80 μM photons m^−2^ s^−1^; as the light level decreases, the nitrogen balance within the fronds is disrupted, leading to non-absorption and even excretion of ammonium [37]. Light intensity in the Yellow Sea varies between 60 and 140 μM photons m^−2^ s^−1^ during May to June, which can thus fully satisfy the growth of *U. prolifera* [9].

### Monsoons and ocean currents drive the northward migration of *U. prolifera*

The southeastward monsoons prevail in the Yellow Sea from April to July, driving surface ocean currents northwards and thus the northward migration of *U. prolifera* fronds [9,38,39]. Wind direction is one of the most important factors affecting the migration direction of *U. prolifera*. Remote sensing data show that the distribution of *U. prolifera* in surface waters appears as a linear band and is consistent with the main path of monsoons [40]. In the Jiangsu coastal area, the *U. prolifera* fronds that are scraped off the *Porphyra* mariculture rafts or the fragments mixed up from the bottom by upwelling are scattered throughout the surface water [9,37]. These fronds flow through the sand-ridge channels and out to the open sea with the residual tidal currents. When *U. prolifera* reaches the open sea, the dynamics of the algal accumulation are driven by a complex ocean dynamic process, including the southeast monsoons, Ekman drift, Langmuir circulation, Subei and Lunan coastal currents [39–41]. In addition to the main northward path, a new southward path along the Subei coastal current was identified, extending from the Jiangsu coast to the East China Sea [12]. The muddy coastal current with *U. prolifera* fronds reaches the southwestern East China Sea in early May along with severe local eutrophication, leading to the occurrence of green tides in this new area [42,43]. Moreover, in 2009, the wind direction of monsoons was quite different from that in other years, with the southwest monsoon prevailing in the Yellow Sea. Driven by the southwest monsoons, only a very few floating patches of *U. prolifera* could reach the coastline of the Shandong Peninsula [44]. As *U. prolifera* did not pile up at the coastline as usual, instead most was floating in the ocean, so the *U. prolifera* green tides in the Yellow Sea in 2009 had the largest distribution area during the past decade.

## ANTHROPOGENIC PERTURBATIONS AND MACROALGAL BLOOMS

### Increasing atmospheric CO_2_ and global warming promote the photosynthetic carbon fixation of *U. prolifera*

**Figure 3.**
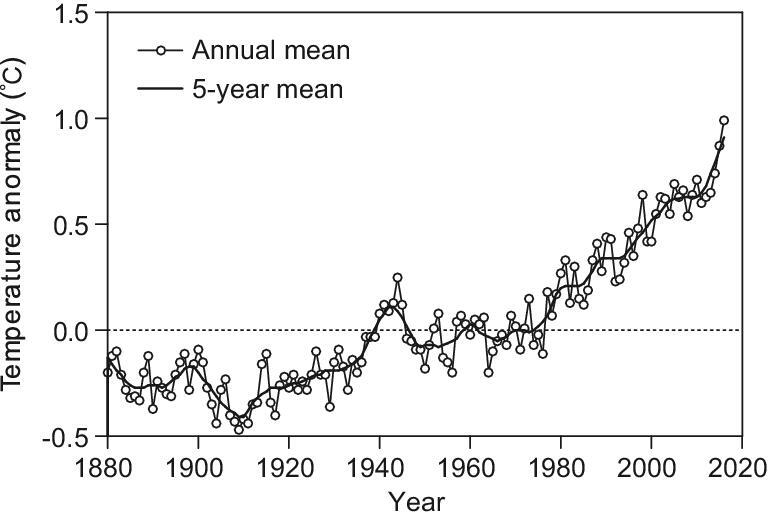
Changes in global surface temperature from 1884 to 2015, as compared to the averaged 1951–80 temperature indicated by the thin dotted line. Source: NASA GISS, https://climate.nasa.gov/vital-signs/global-temperature/.

Global warming has become one of the most important issues worldwide and is mainly attributed to increasing population density and intensive anthropogenic activities [45]. Coastal ecosystems are suffering from various negative effects caused by increasing temperature, which can be embodied as harmful red/green tides, anoxia and storm surges. In recent years, global temperature has increased rapidly and the annual global surface temperature anomaly has increased by 0.6°C since 2008, as compared to the average temperature from 1951 to 1980 (Fig. 3). Meanwhile, global CO_2_ concentration is also increasing. In October 2016, the World Meteorological Organization (WMO) in Geneva claimed that the global average CO_2_ concentration reached 400 ppm for the first time in 2015 [46]. In 2017, the atmospheric concentration of CO_2_ reached 406 ppm according to the latest WMO dataset. In fact, both the rising temperature and atmospheric CO_2_ facilitate the growth and photosynthetic carbon fixation of phytoplankton. Thus, we inferred that rising atmospheric CO_2_ and continuous global warming primarily caused by anthropogenic activities are likely to be among the important factors that facilitate the early occurrence and continuous expansion of green tides. Ocean acidification has a typical negative impact on the increasing of atmospheric CO_2_ and global warming. Laboratory studies have indicated that ocean acidification and warming could accelerate the onset and magnitude of gamete settlement and increase the reproduction of *Ulva rigida*—a causative green-tide species closely related to *U. prolifera* [47]. Based on the model, it is also predicted that the bloom-forming *Ulva* would dominate coastal regions as populations of non-blooming species would decrease faster and maintain lower growth rates, even under mild carbon enrichment [48]. Thus, climate changes are considered to be possibly responsible to some extent for more severe green tides, particularly in a scenario in which eutrophication cannot be effectively controlled [47].

### Increasing nitrogen levels support the growth of *U. prolifera*

Green macroalgae blooms are occurring more frequently and the magnitudes of the blooms are getting larger worldwide. In recent decades, the Baltic Sea, Knysna Estuary, South Africa, Biscayne Bay, Narragansett Bay, South Korea and Japan coasts all have suffered from massive macroalgal blooms [46,49–55]; Brittany (France) has even been experiencing large-scale macroalgal blooms annually since the 1970s [56], which are closely associated with coastal eutrophication [57].

**Figure 4.**
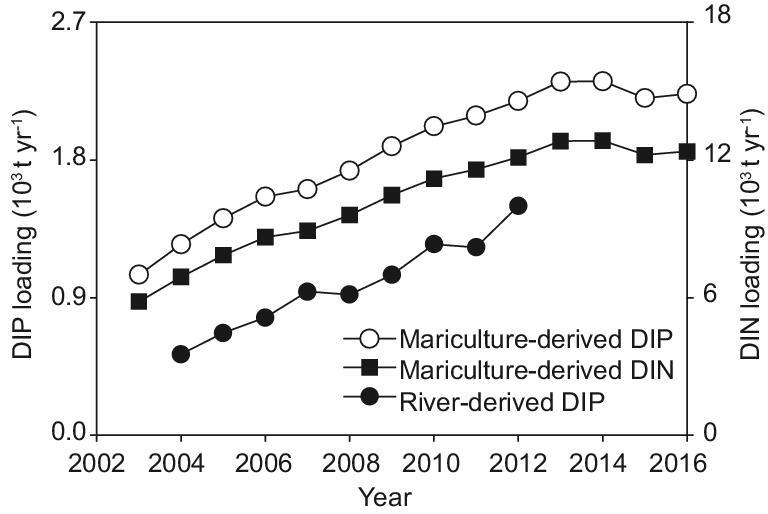
Nutrient loadings from river inputs and aquaculture along the Jiangsu coast from 2003 to 2016. Nutrient loadings from river inputs refer to the sum from the Guanhe and Sheyang Rivers; nutrient loadings from aquaculture are calculated based on yearly aquaculture total production (wet weight) (data between 2003 and 2012 obtained from [71], data between 2013 and 2016 obtained from the Jiangsu Fishery Statistical Yearbook). DIN, dissolved inorganic nitrogen; DIP, dissolved inorganic phosphorus.

With the increase in population and rapid economic development, nutrient loadings have been influenced by anthropogenic perturbations in the coastal waters of the Yellow Sea [16,58]. At present, eutrophication along the Jiangsu coast is becoming more and more serious. The area-weighted nutrient pollution (AWCPI-NP) index of this area for 2007–12 increased by 45% compared to that of 2001–06 (*P* < 0.05) [59]. The Sheyang and Guan Rivers are the two largest rivers flowing through Jiangsu Province. The annual N and P loadings of these two rivers into coastal waters between 2007 and 2012 were approximately three to five times higher than those between 2004 to 2006 [16]. Moreover, the Jiangsu coastal area is the largest shrimp and crab aquaculture zone in China, with thousands of aquaculture ponds along the coast. Over 50 000 tons of fertilizers such as fermented chicken manure, which is rich in organic N and P, are poured into the ponds yearly [60–62]. Discharge of nutrient-rich wastewater from these ponds supplies abundant nutrients to near-shore waters. As shown in Fig. 4, the N and P loadings markedly increased from 2003 to 2016 due to both river inputs and aquaculture wastewater discharge along the Jiangsu coast, which contributed greatly to the eutrophication of this coastal area. Besides the Yellow Sea, the Gouqi Island (in the East China Sea) underwent a *U. prolifera* green tide for the first time in 2012, indicating a close relationship between *U. prolifera* green tides and severe local eutrophication [43].

**Figure 5.**
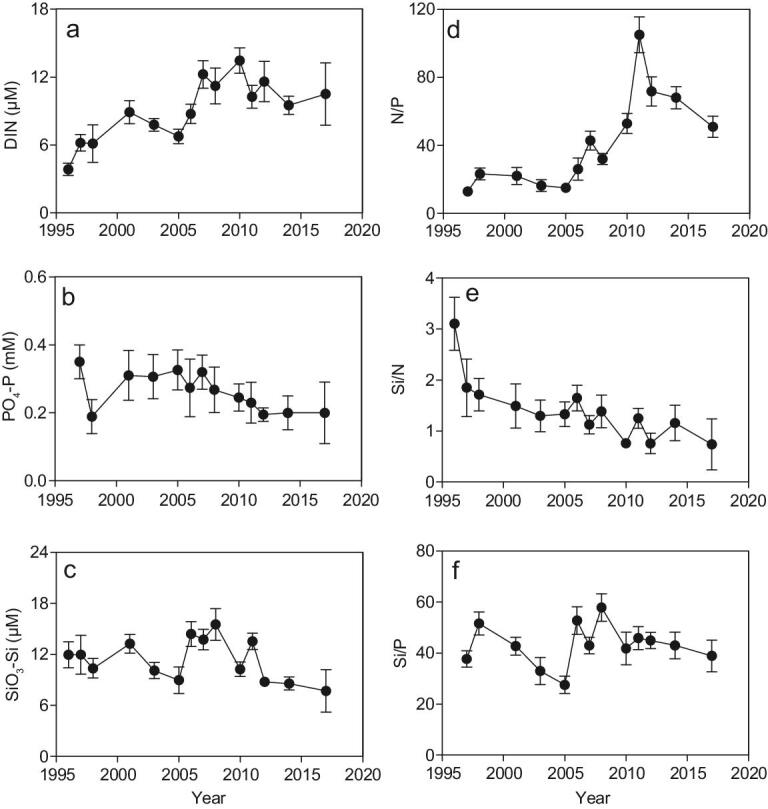
Variations in DIN (a), PO_4_–P (b) and SiO_3_–Si (c) concentrations and N/P (= DIN/PO_4_–P) (d), Si/N (= SiO_3_–Si/DIN) (e) and Si/P (= SiO_3_–Si/PO_4_–P) (f) ratios in the surface waters (0–0.5 m) of the Jiangsu coast. The data were collected from March to May (data between 1996 and 2014 were obtained from [71]; there are no data during 2015–16; data of 2017 were obtained from field surveys).

Nitrogen and phosphorus are two essential nutrients for the growth of *U. prolifera* [63,64]. The maximum growth rate of *U. prolifera* can reach 56% day^−1^ with sufficient N and P supplies [4]. Dissolved inorganic nitrogen (DIN) is particularly critical for the growth of *U. prolifera*. Based on a mesocosm experiment, *U. prolifera* fronds can grow vigorously by consuming DIN quickly, even though the P concentration is fairly low in seawater [64]. Lianyungang, Yancheng and Nantong are three coastal cities situated along the Jiangsu coast where DIN concentrations vary from 10 to 80 μM. The DIN values are only 0.80–5.8 and 0.70–16 μM in the Yellow Sea and in Qingdao coastal waters, respectively [5]. Based on nine surveys conducted between March and September 2012, DIN concentration in the surface water gradually decreased with the green-tide bloom, whereas the lowest DIN level was still over 7.0 μM in the coastal waters of Jiangsu Province [58]. Moreover, the average DIN concentration in the surface water has increased by two to three times along the Jiangsu coast since 1996 to the most recent 3 years (Fig. 5a), indicating that the level of N is sufficient for macroalgal blooms [2]. The PO_4_–P concentration showed a decreasing trend during the study period (Fig. 5b) and the unbalanced changes in N and P caused a significant increase in the N/P ratio during 2005–14 (Fig. 5d). *Ulva prolifera* might adapt well to high N/P conditions and grow vigorously under P-limited conditions [16,64]. There were obvious changes in the silicate concentration and Si/N and Si/P ratios from 1996 to 2017 (Fig. 5c, e, f), but Si is generally thought to be a non-limiting factor for the growth of *U. prolifera* [65].

### *Porphyra* mariculture rafts provide substrata for the macroalgal blooms

**Figure 6.**
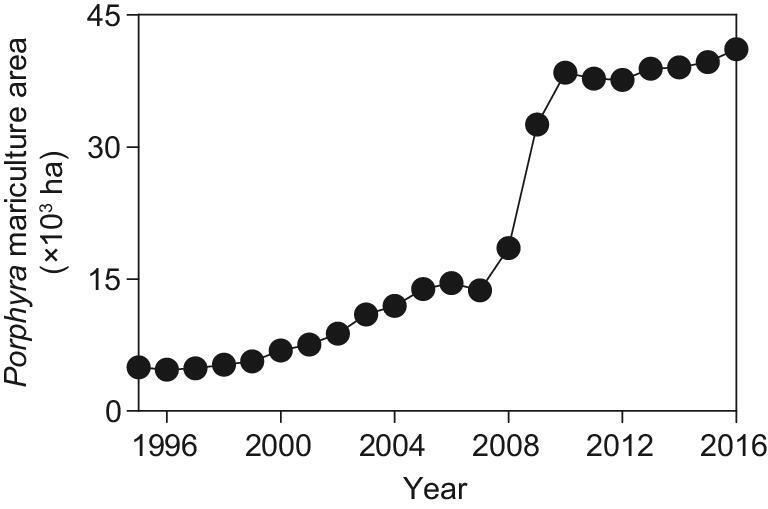
Annual variations in the *Porphyra* mariculture area in Jiangsu Province between 1995 and 2015 (data obtained from the Chinese Fishery Statistical Yearbook).

Besides eutrophication, the rapid expansion of *Porphyra* mariculture is another important anthropogenic factor that contributes to the development of green tides [4]. Jiangsu Province has a long history of *Porphyra* mariculture, with the annual fresh *Porphyra* production reaching approximately 12 600 tons, and it is the second largest *Porphyra yezoensis* mariculture zone in East Asia, closely following Japan [60,66,67]. The annual *Porphyra* mariculture starts in August and ends in the following April, during which massive ropes, bamboo poles and nylon nets are installed in the mariculture regions [59]. At present, the coverage area for *Porphyra* mariculture is approximately 4.1 × 10^4^ ha with about 4500 m of ropes per ha being used for the cultivation, which is thought to provide substrata for *U. prolifera* [16]. When *Porphyra* is harvested from mid-April to mid-May, *U. prolifera* fronds that are attached to the rafts fall into seawater, together with the *U. prolifera* fragments removed from the ropes by local farmers, both of which provide the most direct and initial biomass supply for the green tides [68]. Annually, approximately 6500 tons of *U. prolifera* fall off the *Porphyra* rafts, 62% of which float in the surface water, spread out with wind and ocean currents, and finally bloom into massive green tides [15]. The area used for *Porphyra* mariculture in 2016 was about eight times larger than that in 1995, which clearly increased since the first macroalgal bloom in 2007 (Fig. 6). The sharp increase of *Porphyra* mariculture area in 2007 is possibly one of the most important causes leading to the outbreak of the world's largest *U. prolifera* green tide in 2008. Before 2006, most *Porphyra* mariculture activities were in the intertidal zone near the coastline of Jiangsu Province. The *U. prolifera* that falls from the *Porphyra* rafts could hardly reach the offshore areas due to the reverse movement of tidal currents. However, in the following few years after 2006, the *Porphyra* mariculture expanded very rapidly to the out area of the sand ridges along the Jiangsu coast. These sand ridges are about 200 km long and 100 km wide. The unique radial geomorphology there can frequently induce the formation of upwelling in the sand-ridge channels. Meanwhile, under the driving force of southeast wind, the large amounts of *U. prolifera* fragments falling from *Porphyra* mariculture rafts floated on the surface water and moved northwest. It eventually induced the outbreak of large-scale green tides in the Yellow Sea near the Shandong Peninsula in 2008 [4,5,8,9].

## BIOLOGICAL CHARACTERIETICS OF *ULVA PROLIFERA* CONFER ITS DOMINANCE IN THE YELLOW SEA

Based on its morphology and genomic characteristics, *U. prolifera* is considered as the dominant green-tide-forming seaweed in the Yellow Sea. It accounts for over 90% of green-tide macroalgae, owing to its high nutrient-uptake rate, high photosynthetic capacity and multiple reproduction modes. These biological characteristics endow *U. prolifera* with a strong ability to tolerate changes in seawater temperature, light intensity and nutrient levels during floating. It also owns high growth and reproduction capacities. All these factors might lead to the massive green tides.

### High nutrient-uptake rate

*Ulva prolifera* assimilates inorganic N and P very quickly and has high uptake rates under nutrient-rich conditions [69]. The nitrate- and ammonium-uptake rates of *U. prolifera* (*V*_max-NO3-N_ = 124 and *V*_max-NH4-N_ = 284 μM g^−1^ DM h^−1^, DM refers to the algal dry weight) are higher than those of other *Ulva* species (e.g. *V*_max-NO3-N_ = 109 and *V*_max-NH4-N_ = 250 μM g^−1^ DM h^−1^ for *U. linza*) in Jiangsu coastal waters. Accordingly, the growth rate of *U. prolifera* is higher than that of *U. linza* (*S_U. prolifera_* = 13% d^−1^ vs *S_U. linza_* = 10% d^−1^) [23]. Moreover, eukaryotic microalgae normally cannot survive when co-cultivated with *U. prolifera*, as most of the nutrients in seawater are absorbed and utilized by *U. prolifera* [70]. The maximum DIN uptake rate of *U. prolifera* can be as high as 110 μM g^−1^ DM h^−1^ [71], which makes *U. prolifera* outcompete microalgae by reducing N to levels below which microcells cannot grow [70]. Hence, green and red tides have seldom occurred simultaneously in Chinese coastal waters.

**Figure 7.**
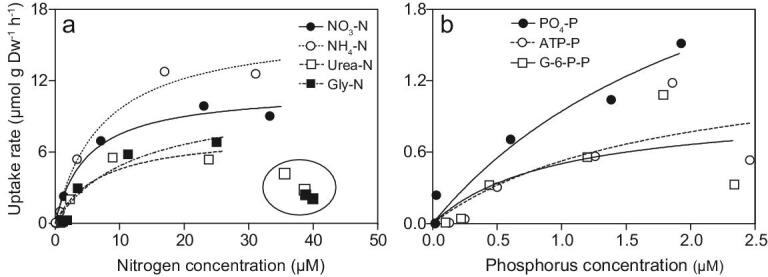
The concentration-dependent uptake rates of (a) dissolved nitrogen and (b) phosphorus of *U. prolifera* during an incubation experiment (modified from [70]). The fitting curves excluded the circled data points.

In addition to inorganic nutrients, some low-molecular-weight organic nutrients like urea, glycine and adenosine triphosphate can be absorbed by *U. prolifera* [71]. The uptake of both inorganic and organic nutrients by macroalgae can be described as a saturating function of substrate concentrations using the Michaelis–Menten equation (Fig. 7). During the early blooms of green tides, *U. prolifera* is most prone to taking up inorganic nutrients and subsequently large amounts of DIN and dissolved inorganic phosphorus (DIP) are utilized in the development of green tides [71]. Hence concentrations of dissolved inorganic nutrients are relatively low during the mid-late development periods of green tides, while the uptake ability of *U. prolifera* to organic nutrients satisfies their nutrient demand and subsequent blooms. Temporal variations of dissolved nutrients were investigated in the southern Yellow Sea during the green-tide-blooming period, and it was found that the DIN decreased rapidly and varied from 21 to 8.0 μM during late April and early May (i.e. early blooms). The DIN decreased to below 5 μM in June and July; simultaneously, the concentrations of dissolved organic nitrogen (DON) began decreasing very fast and varied from 28 to 16.5 μM. Even in late July and August (i.e. late blooms), the DON always maintained ∼15 μM in seawater and the DON provided sufficient nutritional conditions for the growth of *U. prolifera* [72]. Similar results were also observed in another field investigation [58], the concentrations of DON and dissolved organic phosphorus (DOP) decreasing from 31 to 15 μM and from 0.40 to 0.10 μM during the macroalgal blooms. These results indicate that, besides inorganic forms, organic nutrients indeed play an important role in the growth of *U. prolifera* [4,15], especially during the mid-late blooms of green tides.

### High photosynthetic capacity

Most marine algae primarily perform C_3_ photosynthesis. *Ulva prolifera* fronds also have the key enzymes for C_4_ photosynthesis, so it may perform both C_3_ and C_4_ photosynthesis [73,74]. *Ulva prolifera* also has the *lhc SR* gene and non-photochemical quenching mechanism, both of which give *U. prolifera* a high photosynthetic carbon-fixation capacity and environmental adaptability to changing temperatures and light levels [75,76]. Moreover, *U. prolifera* has high photosynthetic plasticity, which may ensure its ability to obtain sufficient energy to increase its biomass and to adapt to a long-distance migration [21]. For example, *U. prolifera* can be periodically transferred from the surface to the bottom of the water column due to vertical mixing when floating from the southern to northern Yellow Sea. When exposed to the surface water, *U. prolifera* fronds can dissipate excess energy via photosynthetic quantum control (energy quenching and energy redistribution between PSII/PSI) to avoid light-caused damages to the photosynthetic system; when mixed into deeper waters, the fronds can promote light utilization by increasing their cellular chlorophyll a (Chl a), chlorophyll b (Chl b) and Chl b/Chl a ratio [21]. The higher photosynthetic capacity of *U. prolifera* contributes to its free-floating proliferation and prevalence among floating green algae species in the Yellow Sea [77].

### Multiple reproduction modes

The life history of *U. prolifera* is very complicated. *Ulva prolifera* has multiple reproduction modes, including sexual, asexual and vegetative propagation [78], among which asexual and vegetative propagation primarily contribute to its rapid expansion within 1 or 2 months. The fronds of *U. prolifera* can grow by (i) increasing algal tubular diameter, (ii) forming new branches, (iii) releasing spores and (iv) expanding fronds [28]. *Ulva prolifera* can release 1.2 × 10^7^ spores or 2.3 × 10^7^ gametes per cm^2^ frond, and 92–97% of them can bind to new micropropagules to germinate [79]. These unique and multiple reproductive methods allow the opportunistic macroalgae *U. prolifera* to maintain colonization and excessive growth, resulting in the formation of massive green tides.

## ROLES OF MICROBIAL ORGANISMS DURING MACROALGAL BLOOMS

Algae are closely associated with bacteria in natural seawater. It is extremely difficult to obtain one sterile alga without using antibiotics or a streaking-based algal separation method with multiple plates. The relationship between algae and bacteria is sophisticated, involving symbiosis, competition, lethality, gene transfer, etc. *Ulva prolifera*, being no exception, is closely associated with microbial organisms. *Ulva prolifera* contains endophytes within and around its fronds. All these microbial organisms may play a vital role in regulating the formation and recession of macroalgal blooms. For example, the morphogenesis of *U. linza*, which is similar to that of *U. prolifera*, depends on the regulation of the key microbial group Cytophaga-Flexibacter-Bacteroides that ensures the normal growth of its fronds [80]. Moreover, the fronds of *U. prolifera* are commonly surrounded by abundant heterotrophic diazotrophs during the blooms [24]. Our previous studies indicate that dissolved organic N could support the growth of *U. prolifera* during the mid or late development stages of green tides [71]. Given that the inorganic N concentration is low in the late stage of green tides, a large number of diazotrophs attached on the fronds may satisfy the organic N demand of *U. prolifera* through N fixation.

Microorganisms have been reported to grow vigorously in seawater at the end of red tides [81]. These microorganisms are generally thought to be a key factor in inducing the recession of red tides and, accordingly, the ‘use of microbial organisms for algal bloom control’ has been proposed [81]. Similarly, massive growth of *U. prolifera* was observed to be related to or to reciprocally have a significant effect on certain microbial communities, and the abundance of microorganisms increased significantly by the end of *U. prolifera* green tides [82,83]. So far, it is still unclear whether or not microorganisms contribute significantly to the recession of green tides in the Yellow Sea. At least, it is certain that microbial activities, via decomposition of the massive dead-and-sunk *U. prolifera* after the green-tide demise, do influence the environment of the Yellow Sea.

## ENVIRONMENTAL IMPACTS OF MACROALGAL BLOOMS

Green tides have profound and long-lasting effects on the environment. In the early and middle development stages of *U. prolifera* green tides, efficient photosynthesis of *U. prolifera* quickly assimilates inorganic carbon, which supports its growth, thus leading to an increase of pH in seawater. The field investigation carried out in 2014 showed that the pH increased up to 8.6 at the centre of a free-floating *Ulva* patch (about 100 000 m^2^) along the Dafeng coast (Jiangsu Province). Massive carbon fixation and abrupt pH increase caused by large green tides may have a significant impact on the phytoplankton population and the community structure and ecosystem functions at the regional scale. At the end of green tides (mid-July to early August), massive *U. prolifera* fronds that fail to be salvaged will sink down to the bottom or suspend at certain depths where they die and are decomposed gradually by microorganisms [84]. The decomposition of *U. prolifera* fronds consumes a large amount of oxygen, resulting in low-oxygen or even hypoxic conditions in the coastal waters. Hypoxia can not only cause massive deaths of cultured organisms (e.g. sea cucumbers and abalone) and induce considerable economic losses, but also affect benthic community structure, biomass and inhabitant density [85,86]. Simultaneously, the decay of *U. prolifera* fronds can release abundant C, N and P, lowering the water quality and affecting the reproduction and respiratory metabolism of heterotrophic microorganisms [53]. Accordingly, large amount of CO_2_ will dissolve into seawater, decreasing the pH of seawater and accelerating coastal acidification, which are well known to have a profound impact on marine ecosystems [87,88]. As shown in the two surveys conducted in the Yellow Sea in 2008, the dissolved inorganic carbon concentration and sea–air CO_2_ partial pressure (*p*_CO2_) increased markedly while the pH decreased substantially during the late-bloom period (22–25 July 2008) of green tides, indicating that coastal waters become a carbon source after a green tide [89]. Owing to microbial activities, decomposition of *U. prolifera* releases substantial dissolved organic matter into the surrounding waters, promoting the growth of microbial organisms, which in turn accelerates the decomposition of dead *U. prolifera*. Accompanied with this process is the release of more CO_2_ driven by microbial respiration, resulting in worse coastal acidification. On the other hand, the released dissolved organic carbon (DOC) during *U. prolifera* decomposition is partially transformed into refractory DOC (RDOC) via microbial metabolism according to the microbial carbon pump theory [90–93]. The RDOC is hard for microorganisms to utilize or degrade, and thus accumulates in the water for a long time, which contributes to carbon sequestration in the ocean.

On 23 July 2016, in the late development stage of a green tide, massive *U. prolifera* fronds floated into the coastal waters of Qingdao, with the largest coverage area in the Golden Beach waters (120°14′38′E, 35°57′25′N), followed by Tuandao (120°18′22′E, 36°03′07′N), Maidao (120°25′47′E, 36°3′29′N) and Shilaoren (120°29′9′E, 36°5′40′N) near-shore waters. We monitored the water physiochemical parameters in the above four waters and found the lowest seawater pH and dissolved oxygen (DO) appeared in the Golden Beach near-shore waters, along with the highest chemical oxygen demand (COD), DOC and bacterial abundance. The DON concentration in this area was similar to that in the Maidao near-shore waters (Table 1). When compared to the seawater quality during the pre-bloom period of green tides (pH: 8.1, DO: 6.3 mg L^−1^, COD: 2.2 mg L^−1^), in the Golden Beach, the pH and DO decreased by 7 and 68%, respectively, whereas the COD during the post-bloom period was 22 times higher than that of the pre-bloom period (Table 1). These results indicate that a great mass of *U. prolifera* in the surface water greatly influences the seawater quality and even causes serious negative environmental effects such as coastal hypoxia and acidification.

**Table 1.** The pH, concentrations of dissolved oxygen (DO), chemical oxygen demand (COD), dissolved oxygen carbon (DOC) and dissolved organic nitrogen (DON) and bacterial abundance (mean ± SD from triplicate samples) in the four sampling sites along the Qingdao coastal areas.

						
Sampling	pH	DO	COD	DOC	DON	Bacterial abundance
sites		(mg L^–1^)	(mg L^–1^)	(mg L^–1^)	(mg L^–1^)	(×10^6^ cells mL^–1^)
						
Golden Beach	7.6	2.0	48.9	13.8	0.5	10.5
	±0.03	±0.2	±1.5	±1.2	±0.08	±1.6
Tuandao	8.2	6.8	25.6	3.6	0.2	4.8
	±0.02	±0.1	±1.2	±0.6	±0.05	±0.7
Maidao	8.0	8.0	36.7	10.8	1.3	12.8
	±0.01	±0.2	±1.9	±0.8	±0.03	±1.3
Shilaoren	8.4	7.2	33.3	5.2	0.2	4.6
	±0.02	±0.2	±0.5	±0.6	±0.06	±0.3

The decomposition of *U. prolifera* releases high concentrations of transudates that may inhibit the growth of some phytoplankton species; conversely, these transudates containing abundant N and P may enhance the growth of some other opportunistic species. When *U. prolifera* is co-cultured with microalgae, the microalgae growth, especially for diatoms, is generally inhibited by monopolizing nutrients or toxic exudates from *U. prolifera* [94]. Nevertheless, substantial amounts of nitrogen nutrients, mainly ammonium, are released after *U. prolifera* decomposition, which is likely to cause regional eutrophication and promote the growth of red tide species, especially dinoflagellates and raphidophytes [95,96].

### WHY DID GREEN TIDES DECREASE IN 2017?

In 2017, the *U. prolifera* blooming scales shrank sharply compared to those in the previous years, which could be attributable to several aspects. Shrinkage of aquaculture due to worsening water quality and increasing disease may be one of the main reasons [97], since such shrinkage can decrease the nutrient inputs into the Jiangsu coast where *U. prolifera* green tides originate [14]. Adjustment of the *Porphyra* harvest mode and time in 2017 (i.e. the harvest time was earlier than before) may also account greatly for the green-tide shrinkage in Qingdao, as it can decrease the amount of *U. prolifera* released into the water. Moreover, during this period, ‘golden tides’ of *Sargassum* blooms, another macroalgae, occurred in the Yellow Sea, covering an area of over 47 713 km^2^ under uncommon currents and wind conditions [98]. The *Sargassum thallus* has well-developed gas bladders and differentiated parts resembling leaves and stems, giving it a competitive advantage for space and nutrients compared to the *Ulva* thallus, thus inhibiting the growth of *U. prolifera*. Moreover, the floating *Sargassum* golden tide occurred in the *Porphyra* aquaculture area, resulting in the decrease of attached *U. prolifera* seaweed and further the shrinkage of the *U. prolifera* green tide during the year.

Furthermore, *U. prolifera* is an edible macroalgae, as its protein content reaches as high as 35%. In recent years, *U. prolifera* has been considered good material for foods and the economic benefits thus incentivize local freshmen to widely collect the attached or floating *Ulva* seaweed in the Yellow Sea. Such *Ulva* collection would finally decrease the number of floating blooms in the northern Yellow Sea.

In addition, the abnormal weather conditions caused by the El Niño phenomenon were likely related to the reduction of the *U. prolifera* green-tide scale in 2017; the El Niño-caused decrease in precipitation and increase in temperature may respectively lower the levels of nutrients that are derived from the land (e.g. in 2017, there was more drought during January to April than ever before in Jiangsu and Shandong Province) or from the stratified bottom layer [99]. Furthermore, El Niño might have altered the marine hydrodynamics, such as weakening the northward currents (e.g. the Subei coastal current) that typically drift the *U. prolifera* fronds northward [14].

During 2018, the maximum distribution area of green tides was reported to be 34 097 km^2^ in the southern Yellow Sea (Fig. 1, data of 2018 were from the website of North China Sea Branch of State Oceanic Administration). Though the macroalgal bloom scale exceeded that of 2017, the outbreak scale of green tides in 2018 was much smaller than those of previous years (i.e. 2014–16). The shrinkage of aquaculture along the Jiangsu coast in recent years and the adjustment of *Porphyra* harvest mode and time were potentially two main reasons for the shrinkage of green tides during 2017 and 2018.

## POTENTIAL CONTROL METHODS FOR GREEN TIDES IN THE YELLOW SEA

### Reduction of nutrient input from diverse sources

Nutrient inputs from diverse sources are considered to strongly influence the reoccurrence of *U. prolifera* green tides in the Yellow Sea. For a truly integrated management of the coastal zone, reduction in nutrient input and control of effluents, especially for N-source nutrients from the coastal pond systems, are needed to control eutrophication and prevent green tides in the future.

### Setting intercept lines to control the spread of the green tides in the Yellow Sea

Controlling the green tides in the source region would be the most effective method to eliminate the outbreak of green tides. Generally, the initial blooms will take 30–40 days to drift to the northern Yellow Sea. Thus, it would be better to set one intercept line in the origination sea area to stop the northward migration of *U. prolifera*. Another intercept line could be set in the coastal area of the Rizhao district, which is next to the southern coast line of Qingdao city, so as to clean the scattered floating fronds that may escape the first intercept line. These two intercept lines would play a key role in preventing the spread of the green tides in the Yellow Sea. Besides, the collected *Ulva* seaweeds can be processed into valuable products.

### Compensation mechanism for harvesting *U. prolifera*

It has been suggested that more hygienic harvesting practices for *P. yezoensis*, such as land disposal of *Ulva* species on the bamboos and ropes, should be adopted to prevent the recurrence of green tides in the Yellow Sea [8]. Further, if a high biomass of floating *Ulva* species was collected before the two intercept lines mentioned above, the negative impact of green tides on the coastal ecology and economy of the Yellow Sea would be greatly reduced. However, due to the low value of *Ulva* species compared to *Porphyra* and other aquatic animals, farmers would not want to collect a high biomass of the *Ulva* species that are attached on *Porphyra* rafts and floating on the sea surface. Therefore, it would be better for governmental policy makers to set up a compensation mechanism for the harvesting of *Ulva* species. For example, farmers can collect *Ulva* species attached or floating biomass and sell them to manufacturers at a certain price to produce food, feed and other products.

In addition, the earlier harvest of *Porphyra* in the southern Jiangsu Province, especially before the temperature increases to over 10°C in April, might reduce the biomass of *U. prolifera* dropping off the rafts and thus shrink the outbreak scale of the green tides. This is because temperature has an important impact on the growth of *U. Prolifera*; for example, *U. prolifera* can exhibit normal photosynthesis only if the temperature is higher than 10°C [26,27]. A reasonable and scientific compensation mechanism is very important for the farmers to stay positive.

### Comprehensive utilization of the huge biomass of green-tide algae

*Ulva prolifera* is a natural biotic resource. In Zhejiang and Fujian Province, *U. prolifera* has been treated as a delicious food that can often be found in the market. However, the current utilization of *U. prolifera* is relatively simple, mainly as fertilizer, feed, food, additives, etc. It is of great significance to further develop and utilize *U. prolifera* [100]. Turning the massive *U. prolifera* ‘waste’ into ‘treasure’ not only benefits the local economy, but also helps to reduce the negative impact on the ecological environment.

## CONCLUDING REMARKS

The annual outbreak of *U. prolifera* green tides in the Yellow Sea since 2007 has been attracting worldwide attention. Even though *U. prolifera* fronds are considered to be a natural asset, which could be used to produce organic fertilizer, polysaccharides, etc., their serious negative effects on local mariculture, tourism and environment have caused heavy economic losses. The monitoring and extensive studies in the past decade have shown that the outbreak mechanisms of *U. prolifera* green tides are far more complicated than expected. Multiple factors have been proven to induce the outbreak of large-scale green tides in the Yellow Sea, including regional physicochemical conditions (e.g. temperature, salinity, light intensity, monsoons and ocean currents), algal physiological advantages (e.g. high nutrient-uptake rate, high photosynthetic capacity and the coexistence of multiple reproduction modes) and human activities (e.g. nutrient discharge, CO_2_ emissions), etc. In addition, it has been gradually realized that climate changes, aquaculture modes and biological interactions (e.g. with both microorganism and other macroalgae) also significantly affect the occurrences of *U. prolifera* green tides in the Yellow Sea. Moreover, the consequences of green tides are serious. In the short to medium term, green tides can degrade water quality, increase the outbreak risk of red tides and cause coastal hypoxia and acidification, while, in the long term, they may have a long-lasting impact on coastal carbon cycles. The identification of the outbreak mechanisms of green tides and the following consequences could provide implications for the corresponding countermeasures to be adopted to avoid future occurrences of green tides in the Yellow Sea, and provide reference for the future in ecosystem management and sustainable development.
